# ﻿Ecology and Conservation of the Dutch Ground Beetle Fauna - Lessons from 66 Years of Pitfall Trapping: ﻿By Hans Turin, D. Johan Kotze, Stefan Müller-Kroehling, Pavel Saska, John Spence and Theodoor Heijerman. Wageningen Academic Publishers, 2022, 452 pages. eISBN: 978-90-8686-921-3; ISBN: 978 90-8686-369-3 https://doi.org/10.3920/978-90-8686-921-3. Euro 99.

**DOI:** 10.3897/zookeys.1118.91192

**Published:** 2022-08-24

**Authors:** Dietrich Mossakowski

**Affiliations:** 1 University of Bremen, Bremen, Germany University of Bremen Bremen Germany

## ﻿

Carl H. Lindroth was the first to present a comprehensive survey with his fundamental work on the Fennoscandian carabid beetles, covering all ecological aspects. The knowledge of the Dutch carabid beetle fauna is also exceptionally good what is especially thanks to Piet den Boer’s initiative and the work of his followers.

Apart from the comprehensive book by Hans Turin (2000) on The Netherlands carabid beetles, I only see major publications on parts of a country or certain aspects, respectively, such as Trautner et al. (2017) on carabids of a German Federal State, Baden-Württemberg or Paill (2009) on endemic species in Austria.

Turin et al. (1991) analysed the data bank on Dutch carabid beetles on the basis of 1.616 year samples from the period 1953–1983. The present book contains updated and extended analyses of the extraordinary huge data bank on Dutch carabid beetles of 4.363 pitfall trap year samples with more than 3 million specimens covering additionally the period 1984–2018.


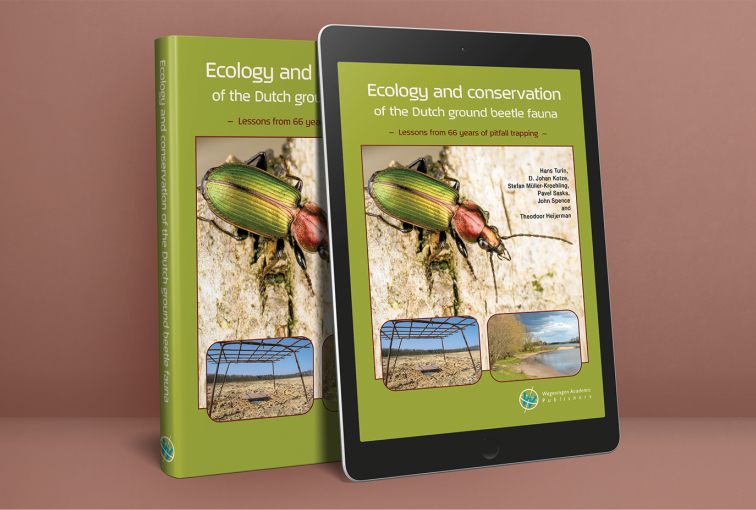
 **The authors**

This book was produced through exemplary teamwork. This makes it difficult to briefly describe the individual contributions; in the book, this cooperation is described in detail on half a page. Hans Turin developed the concept of the book, created most of the text, the figures, the tables, and most of the habitat photos. He also organised the cooperation of the team. The other authors contributed parts of the methodology, parts of the text, habitat and species photos, and read critically and discussed the manuscript.

**Chapter 1 Introduction to the chapters** provides a very helpful, concise summary of the contents of the remaining chapters with references to the relevant paragraphs. It also contains two substantial maps of The Netherlands with the locations of the traps and the hand catches, respectively, which clearly show both the hot spots of the investigations and the geographical gaps.

In general, the text of the whole book is optimally supplemented by impressive figures and tables and is best illustrated by very good habitat photos and mostly perfect photos of the beetles. All are optimally placed in relation to each other.

**Chapter 2 Carabidology** provides a general introduction to the biology and ecology of carabids in particular the aspects relevant to the evaluation of pitfall trap sampling. This chapter represents a revision and update of what is contained in Turin’s (2000) book.

The comparison of the numbers of beetles caught by hand versus those sampled with pitfall traps is very revealing (P. 38). Somewhat complicated to acquire at first glance, but is made easy by the original catch figures of the 12-year-samples shown in the tables nearby.

Heijerman et al. (1989) showed that the placement of pitfall traps is actually more important than the number of traps in order to obtain the most complete list of carabid species present (P. 55).

According to Hutchinson (1959), the ecological niche is the hyperdimensional space of a species’ habitat. Following this concept, a niche cannot be occupied and speciation is niche development (P. 46).

The figures 2.24 and 2.25 (p. 48, 49) are incomprehensible to me. The x-axis has 120%, and some of the bars are placed on the x-axis above 100%. These graphs were taken in their original from Desender (1989).

Although in the references, the work of Lindroth (1946) on the heritability of wing dimorphism is not mentioned here (P. 48).

There seems to be a contradiction: *Carabusnemoralis* is represented in table 2.5.b as found only on the mainland, in the text (p. 74, last paragraph) this species is mentioned for the NO-Polder. In consequence, the discussion about the inheritance of the ability to fly might become obsolete.

An interesting aspect considering phenology/development may be added: in mark-recapture experiments, Althoff et al. (1992) found that *Carabusauronitens* can skip nearly an entire generation. As a result of too low temperatures in May, reproduction of most specimens failed and the beetles overwintered a second time. This effect could not have been detected with normal pitfall trapping.

**Chapter 3 Database work and pitfall traps.** This chapter describes the Dutch carabid database and the strengths and limitations of the pitfall trap method.

Two aspects can be highlighted: (i) The plea for a standard pitfall trap even though “…the problem of incomparability of pitfall results … can be solved by applying the correct statistics…” (P. 87). and (ii) the question of what pitfall trap catches reflect: “… most carabidologists have made peace with the fact that their catch is not equivalent to abundance.” (P. 92).

Pitfall trapping in flooded habitats is possible after all (P. 88). Several trapping methods exist that are not susceptible to flooding but operate during low tides (Floating trap Kiel and Bremen, resp.; Air bell trap (see Dormann 2000)).

**Chapter 4 Exploring the database, methods.** Because evaluation methods are important for understanding, they will be briefly described here.

In a former paper, Turin et al. (1991) described the basis for the evaluation of the samples of the database containing the year samples of 1953–1983. They established 33 habitat types (H1–H33) in seven habitat groups and allocated the carabid species to these groups (HAB1).

In the present book, the scheme was rearranged and named HAB2. Habitats were ordered in three levels: (i) 33 habitat types (e.g., # X1–X5 for heathland) that were allocated to (ii) 17 habitat groups (e.g., peat, wet and dry heath), and in level (iii) the carabid species were allocated to habitats in six affinity groups (e.g., heathland species). The basic structure, the habitat types H1–H33 in HAB1 and X1–X33 in HAB2, is identical. But the meta-structure of habitat groups differs significantly in a number of groups and assignments.

After testing other modern statistical methods, the basic structure of the original habitat types was maintained and the habitat types form the basis for all further comparisons. But the habitat groups were reorganised significantly, and their compliance with the European Habitat Directive was indicated.

The allocation of the carabid year samples to the habitat structure resulted in the habitat preference. The data were manipulated as already described in Turin et al. (1991): The data of a year-sample were standardised by calculating the number of specimens per decimetre pitfall edge and year (SDY), SDY was transformed to ln(SDY+1), and the resulting values adapted to relative abundances by setting the highest value of a species to 100%.

The investigation of a sample or groups of samples was done generally by comparing the data in focus with the reference matrix of the 33 habitat types of the HAB1 classification. The results were presented in three ways: (i) As habitat charts of the 33 Renkonen Indices per sample, (ii) As similarity matrix of the Renkonen Indices and (iii) As species ordination and centroids. Heijerman and Turin (1991) found the Renkonen Index as the most suitable of several measures for the relationship between the data of a pitfall sample and the basic HAB1 matrix of habitat preferences. The results are presented in three ways:

Habitat charts. The graphs show the bars of the 33 values (X01–X33, HAB2). The height of the bars indicates the agreement with the preferences of HAB1 and was considered a fingerprint of the sample. The first and second highest bars were used to allocate the sample to one of the 17 habitat groups (GR01–GR17). Studying the habitat charts, the reader of the book should have in mind that the order of habitat types in the habitat charts is X1–X33 sequentially throughout the text but in the habitat groups (HG) the arrangement differs: X29 is coupled with X12 as habitat group GR8 and X24 with X30 as GR14.

Similarity matrices. Similarity matrices are used to compare multiple samples, for example, to show changes over time in certain habitats.

Ordination and centroids. The species and their abundances were presented in a DCA ordination that resulted from the HAB1 classification. Habitat conditions were indicated within the graph, which was compiled from the literature, independent of the habitat classification of the book. The location of a sample or the occurrence of a species within this ordination plot can be shown in centroid plots. The reliability of the method is shown in detail in one example, it is well proven.

Characteristic and accompanying species. The affinity to one or more habitat groups was performed using the procedure described by Müller-Kroehling (2015): *X*^2^ values were calculated in a test of a four-field contingency table containing the number of samples, in which the focus species and all other species are present or not. Species with a striking high value only in one of the 17 habitat groups were classified as primary characteristic species, those with a maximum in two or three habitat groups as secondary characteristics, and all other species as eurytopic. The characteristic species were listed in table 4.11.

The most abundant eurytopic species that occur in several habitat types were denominated as accompanying species if they co-occur with a characteristic species in at least 40% of year samples.

Table 4.11 (P. 132) provides a quick overview of the characteristic and accompanying species of the habitat groups. A full list according to habitat groups is given in Appendix A. The denomination of habitat groups is listed on p. 177.

Affinity groups. The 203 characteristic species established were grouped into the six affinity groups and described in short in chapter 4.6 (P. 135 ff): Heathland (H), Dunes (D), Grassland (G), Forest (F), and Ruderal and Pioneer (R) species.

The discussion of other classifications led to the insight that the larger the area that is covered the weaker the habitat information and the geographical distribution of species has an important effect.

**Chapter 5 Ground beetle fauna of the Netherlands** is the main part of the book. It contains the evaluation with regard to the six affinity groups, the species accounts and an update on the species accounts in Turin’s (2000) book.

The **first** part deals with affinity groups and their relationships to traits, habitat groups, and their distribution across regions, assemblages, etc. The fact that the geographical occurrence of the species plays an important role is vividly demonstrated in the biography sub-chapter 5.9 with an illustration of the geographic occurrence of eight Dutch forest species.

The **second** part deals with the habitat group accounts.

The 33 habitat types are combined into the 17 habitat groups because the overlap of some types was so great that they did not need to be considered separately. Nevertheless, the habitat types were discussed within the groups. The habitats were classified according to their carabid fauna of a total of 4359 year-samples (table 4.10, p. 130). The presentation follows the same scheme for all 17 groups: an assignment to the EU habitat directive, a short description of the habitat types that belong to the respective group, characteristic and accompanying species, habitat reference, and a summary.

They include map(s) of the locations of the affinity group samples, a plot of the centroids of the group, and a table of the affinity values *X*^2^ of the characteristic species for all 17 affinity groups by which the reader can very well understand the assignment of the species to the respective affinity group, a table with traits of the characteristic species, a table of accompanying species in relation to characteristic ones, habitat charts of selected samples, habitat photo(s), and photo(s) of selected species.

Chapter 5.10 covers pages 175–300.

The **third part** of chapter 5 is an update of Turin’s (2000) species account.

I want to address the discussion of Section 4.7 here, the transferability of the Dutch habitat classification to other regions. It has been made clear that a comparison of the entire classification is only possible in individual cases. This is not only due to methodological reasons of comparable studies (e. g. quantitative data instead of quantitative data, areas of different size), but also to the ecological and geographical conditions of the Dutch landscape. This may be illustrated by three habitat groups.

***Salt marshes *.** The carabids of salt marsh habitats contain a group of species highly specific, in consequence, the characteristic species of this group are also found in salt marshes along the North Sea coast of the adjacent countries. However, the zonation within the salt marsh fauna could not be found because no traps could be set up in the flooded areas.

***Forests*.** It was shown that the results considering forests are difficult to transfer to other countries. This is mainly due to two facts. (i) The distribution of some forest species only partially extends into The Netherlands, which is clearly demonstrated by the distribution maps of eight forest species (P. 164–65, fig. 5.22). (ii) “…it seems most likely that the weak similarities between the Bavarian and the Dutch forest faunas result mainly from the anthropogenic distortion of forest habitats in the Netherlands.” With the exception of the forest fauna of southern Limburg (P. 141).

***Peat bogs, Habitat GR01***: “…little is left in a near-pristine condition.” (P. 178), “… the ground beetle fauna on peaty soils includes a small number of typical inhabitants, such as *Agonumericeti*, *A.munsteri*, …” (P. 181). But *A.ericeti* was found as characteristic species of GR03 (Dry heath). Additionally, it was found in all habitat groups (Tab. 5.14 shows values >0 for GR01–Gr17), thus indicating that this species was found in low numbers as well in dunes as in forests, etc.

These findings are quite different from the situation in Northern Germany. I see four reasons that may interpret these differences. (i) Remainders of raised bogs in a near-pristine status do they really further exist nowadays in the Netherlands? It is unlikely that the situation is better than in northern Germany; (ii) Some GR01 habitats were described as very wet renatured locations (P. 179), but these habitats are not comparable with raised bogs, they harbour only a restricted bog fauna, and do not have hummocks, which are a must for overwintering of *A.ericeti* (Främbs 1994); they do not have bog pools where *A.munsteri* lives in the siltation zone; (iii) Both species are known to be able to survive habitat changes for a long time in low numbers, in consequence, the species may be characterised at such locations as a succession relict; (iv) The realised ecological niche may be different at the border of the distribution area of the species considered (Kühnelt’s principle of regional stenoecia).

The number of km-squares (73) differs in the caption of figure 5.34.A (GR01) from that present within the figure (114). The number of samples in figure 5.34.B shows minor deviations from those of the text and in table 4.10, like in six other habitat groups.

**Chapter 6 Trend analysis** begins with an introduction to the trend analyses already available, in general, for The Netherlands and the Carabids in particular. The data were standardised in terms of effort and analysed using the Generalised Additive Mixed Modelling. The evaluation was done for the whole material and separately with selected habitats: Dunes, Heathlands, grasslands, forest and ruderal habitats. To test the validity of the results, calculations were additionally carried out with reduced material.

The global analyses resulted in a weak but significant decline of the mean catch, which added up to 47% in 2018 to that observed in 1970. The global analysis of species richness did not indicate a change.

The results of the six affinity groups differ from group to group as well considering catch numbers and species richness. Trends were found for heathlands, grasslands, and forests. Several characteristic heathlands species showed a slow but continuous decline in catch numbers that were interpreted as caused by nitrogen deposition from farmland. A low but significant decline was found in catch numbers of forests, while their species richness remained stable. They found a significant decline for non-characteristic grasslands species but not for characteristic species, a fact that is discussed in the light of fertilisers input, vegetation changes and management measures.

**Chapter 7 Conservation.** The importance of carabids for nature conservation and their various threats are described. The chapter emphasises that the focus of conservation should not be on the individual species, but on the communities. “The work of this book is clearly focussed on the relationship between species and habitat and specially between typical communities (habitat groups) and habitats.” In consequence, the usefulness of red lists for conservation management is regarded as limited. The habitat reference method as used in the book was applied to demonstrate by three examples its usefulness for conservation purposes.

An agricultural index was calculated to determine the agricultural influence on the carabid fauna. It is the sum of the *X*^2^ values of the habitat types X12 (farmland intensive, sandy soils) plus X26 (farmland intensive, clay soils) and demonstrated in charts with 14 classes strongly demonstrating the agricultural influence.

The habitats most threatened in The Netherlands are the heathlands and the forest of the hills of southern Limburg.

**Chapter 8 General summary and conclusions.** A summary of the book’s content on one page.

**References.** A compendium of 35 pages of (mainly) carabid literature.

**Appendices.** Species list. It contains the species number in Turin (2000), scientific name, its abbreviation, wing development, reproductive period, geographical position, ecology according to Lindroth (1949), affinity group, numbers of pitfall samples with the species, the same number incl. other sample methods and the number of specimens.

Data contributions list the samples with number, year, province, kind of object (Habitat chart, figure, photo, plot of centroids, table), source, chapter and figure or table number, resp.

Terms list. In this list, 70 terms frequently used in the book were explained.

**Index.** Five pages covering keywords and their most important section of occurrence.

I only found 18 typing errors in the whole book, but they do not affect the understanding.

## ﻿Conclusion

The book presents the successful approach to evaluating the enormous amount of data on the Dutch carabid beetles. The methods used are not only described but their applicability is tested and their selection justified in detail. The naming and labelling are clear and well- arranged and consistent throughout the book. The presentations and results are supported in detail by tables and illustrations and illustrated by impressive habitat pictures and photos of the species, especially the excellent live photographs by Heijerman.

Whereas in Turin (2000) the species is the focus of the presentation, here it is the habitat, the habitat group, the characteristic species and the communities that are the focus of interest.

Besides pitfall catches, the data set takes also into account hand samples. This could only be managed by considering year traps (catches at a minimum of eight weeks in spring plus eight weeks in summer/autumn), which have the advantage to correspond most closely to the actual abundances.

Tables and figures are very well arranged in relation to the text. It is very helpful that not only the page numbers are available in the header, but also the detailed naming of the chapters with numbers and text, so that you always know exactly where to find what.

Among many other interesting studies, the Dutch carabidologists were very good at using the polders of the IJsselmeer and Lauwersmeer, which had been created by diking, for their research. Chapeau!

I strongly recommend putting this volume next to Turin (2000).
